# Dupilumab enables glucocorticoid withdrawal in refractory multisite mucous membrane pemphigoid: a case report

**DOI:** 10.3389/fmed.2026.1915046

**Published:** 2026-08-12

**Authors:** Yuan Hu, Liu-Li Xu, Ke-Han Li, Chun-Shui Yu, Shao-Bo Wang

**Affiliations:** 1 Department of Dermatology, Suining Central Hospital, Suining, Sichuan, China; 2 Department of Gastrointestinal Surgery, Suining Central Hospital, Suining, Sichuan, China

**Keywords:** biologic therapy, dupilumab, IL-13, IL-4, mucous membrane pemphigoid, type 2 inflammation

## Abstract

**Background:**

Mucous membrane pemphigoid (MMP) is a mucosal-predominant, potentially scarring autoimmune subepithelial blistering disease. Although dupilumab is approved for adult bullous pemphigoid, its use in MMP remains off-label and published experience is limited.

**Case presentation:**

We report a 77-year-old man with refractory mucous membrane pemphigoid presenting with recurrent painful oral erosions, ocular symptoms, suspected nasopharyngeal/upper-airway involvement, and widespread cutaneous blisters. Histopathology showed a subepidermal blister, and direct immunofluorescence revealed linear C3 and IgG deposition along the basement membrane zone; BP180 and BP230 antibodies were elevated. Despite long-term systemic glucocorticoids, disease activity persisted, with chronic obstructive pulmonary disease, prior prostate cancer, and 7.5-kg acute weight loss. Dupilumab was initiated with scheduled prednisone tapering. Pruritus and new blister formation rapidly decreased, erosions re-epithelialized, and oral symptoms, ocular dryness, and tearing improved, prednisone was discontinued after 3 months, and dupilumab monotherapy maintained remission without serious adverse events.

**Conclusion:**

This case highlights dupilumab-mediated IL-4/IL-13 blockade as a potential steroid-sparing strategy for carefully selected patients with refractory multisite MMP in whom conventional broad immunosuppression carries substantial risk, while emphasizing that control of active inflammation may not reverse established cicatricial damage and that longer-term controlled studies are needed to confirm its efficacy, durability, and safety.

## Introduction

Mucous membrane pemphigoid (MMP), historically termed cicatricial pemphigoid, represents a heterogeneous group of chronic autoimmune subepithelial blistering diseases that primarily affect mucosal surfaces. The oral cavity and ocular surface are most commonly involved, although lesions may also occur in the nasal, nasopharyngeal, laryngeal, esophageal, and anogenital mucosae; a subset of patients develop cutaneous involvement. The clinical importance of MMP lies not only in blistering and erosions, but also in its potential for irreversible tissue damage. Persistent mucosal inflammation can lead to fibrosis and scarring, resulting in symblepharon, airway narrowing, dysphagia, sexual dysfunction, or permanent visual loss. Early recognition and durable disease control are therefore essential (1–4).

Diagnosis of mucous membrane pemphigoid requires careful clinicopathological correlation, integrating a predominantly mucosal phenotype with subepithelial or subepidermal blister formation, linear deposition of immunoreactants along the basement membrane zone on direct immunofluorescence, and, when available, serological assessment of relevant target antigens, including BP180/collagen XVII, BP230, laminin 332, *α*6β4 integrin, and type VII collagen (2–5). Patients with severe ocular, laryngeal, esophageal, genital, or rapidly progressive multisite disease generally require systemic corticosteroids in combination with conventional immunosuppressants, rituximab, or intravenous immunoglobulin (3, 4, 6). Dupilumab, which blocks IL-4 receptor α and thereby inhibits both IL-4 and IL-13 signaling, has been approved for adult bullous pemphigoid; however, this approval cannot be extrapolated directly to MMP, a clinically distinct, mucosal-predominant, and scar-forming disorder for which evidence remains limited to isolated case reports. Against this background, the present case adds clinical evidence that dupilumab-based therapy may facilitate glucocorticoid withdrawal in an older patient with refractory multisite MMP involving the oral and ocular mucosae, suspected upper-airway disease, and extensive cutaneous lesions.

## Case presentation

A 77-year-old man was admitted with recurrent oral erosions for more than 2 years and worsening generalized blistering for 1 month. His medical history included hypertension for more than 10 years, chronic obstructive pulmonary disease treated with umeclidinium/vilanterol inhalation, and prostate cancer treated with surgery and chemotherapy more than 10 years earlier. Two years before admission, he developed painful erosions of the lips and oral mucosa without an identifiable trigger. Histopathological examination and direct immunofluorescence at an outside hospital were reported to support a pemphigoid-spectrum autoimmune blistering disease. The lesions improved transiently but repeatedly relapsed despite outpatient treatment, including long-term oral prednisone at approximately 0.5 mg/kg/day.

During the same 2-year period, he underwent surgery for nasopharyngeal mucosal lesions and subsequently developed nasal adhesions, breathing discomfort, and hoarseness, raising concern for scar-forming nasal/nasopharyngeal and upper-airway involvement. Approximately 1 year before admission, ocular itching, dryness, tearing, and blurred vision developed and were diagnosed elsewhere as chronic conjunctivitis. One month before admission, multiple tense blisters appeared on the trunk and rapidly ruptured, leaving extensive erosions, exudation, crusting, pruritus, and pain. Escalation of prednisone to approximately 1 mg/kg/day did not prevent new lesions on the scalp and extremities. During this flare, appetite and sleep worsened and body weight decreased by 7.5 kg. He denied a personal or family history of autoimmune blistering disease.

### Diagnostic assessment

**Figure 1:**
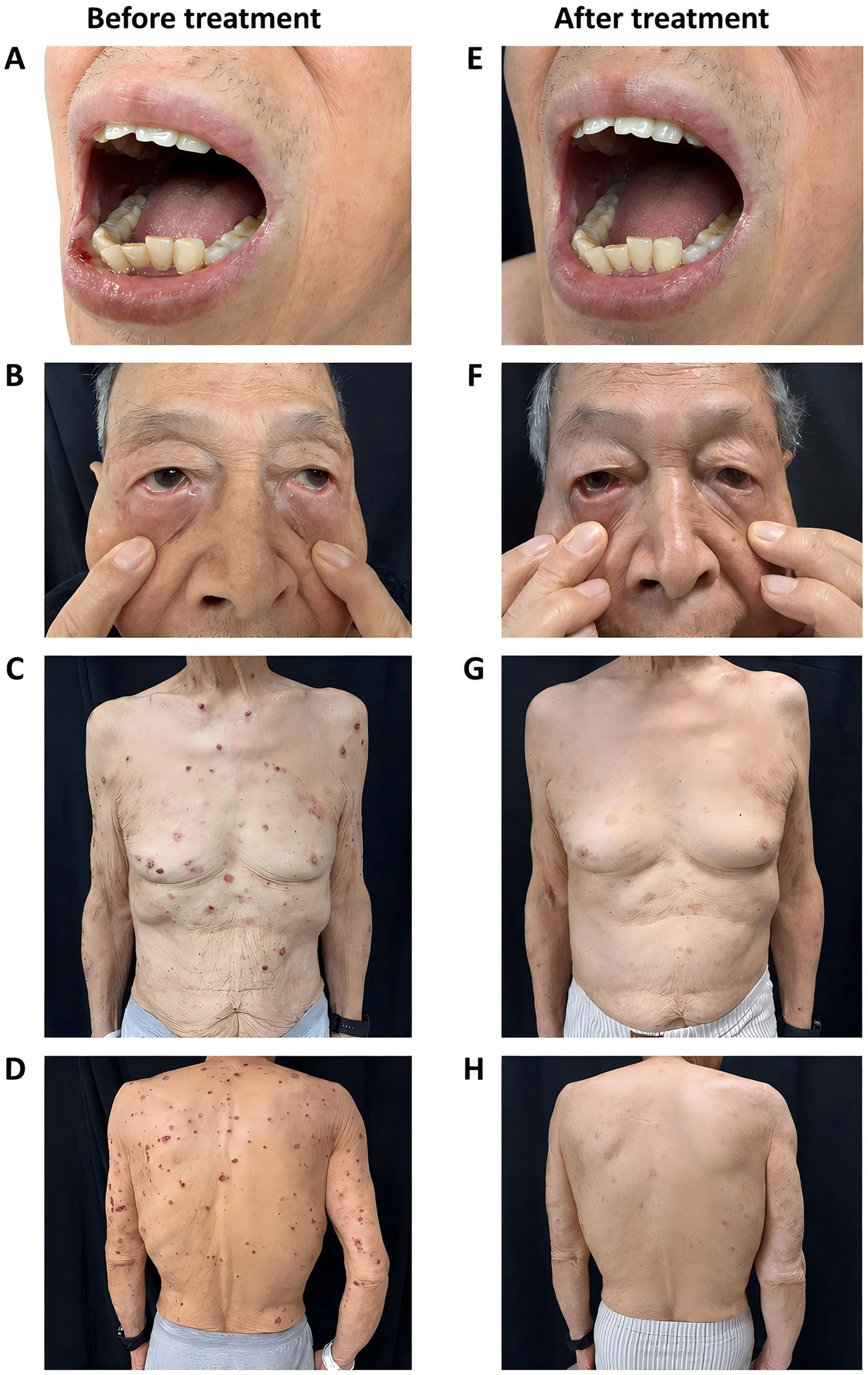
Clinical manifestations before and 1 month after dupilumab-based therapy. **(A–D)** Pretreatment findings showing painful oral erosions and ulcerations, ocular involvement with visible conjunctival adhesion consistent with symblepharon, and widespread blisters, erosions, and crusting on the anterior and posterior trunk. **(E–H)** Findings 1 month after dupilumab initiation showing improvement in oral lesions, ocular dryness, and tearing, markedly reduced blistering and erosions, progressive re-epithelialization, and residual post-inflammatory pigmentation. The pre-existing conjunctival adhesion remained visible without clear reversal.

**Figure 2:**
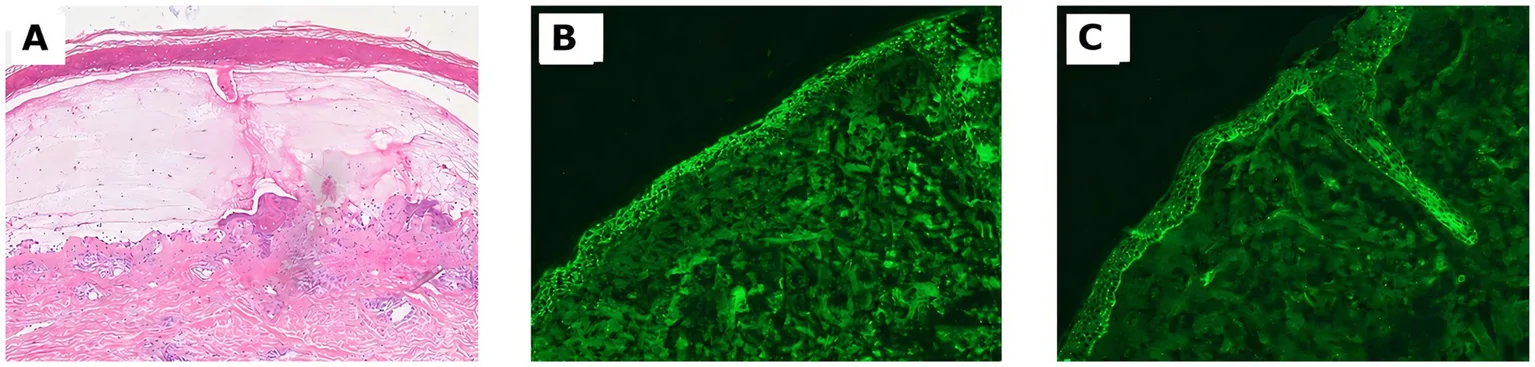
Histopathological and direct immunofluorescence findings. **(A)** Hematoxylin and eosin staining showing a subepidermal blister with inflammatory-cell infiltration. **(B)** Direct immunofluorescence showing linear C3 deposition along the basement membrane zone. **(C)** Direct immunofluorescence showing linear IgG deposition along the basement membrane zone.

At admission, examination showed multiple erosions and ulcers of the oral mucosa and widespread ruptured blisters, erosions, crusts, and exudation on the trunk, scalp, and extremities; Nikolsky sign was negative (Figures 1A–D). Ocular examination and pretreatment clinical photographs demonstrated conjunctival adhesion consistent with symblepharon (Figure 1B), providing visible evidence of cicatricial ocular involvement. Histopathological examination demonstrated a subepidermal blister with inflammatory-cell infiltration, and direct immunofluorescence showed linear C3 and IgG deposition along the basement membrane zone (Figure 2). Serum BP180 and BP230 antibody levels were elevated at 35.83 and 44.53 RU/mL, respectively. Desmoglein 1 and desmoglein 3 antibodies were mildly elevated at 47.77 and 26.64 RU/mL, respectively. Baseline total serum IgE was 125.62 IU/mL, and the absolute eosinophil count was 0.1 × 10^9/L. Serological testing for anti-laminin 332 antibodies was not performed because this assay had not yet been established at our institution; assays for alpha6beta4 integrin and type VII collagen antibodies were likewise unavailable. Histopathology and direct immunofluorescence established a pemphigoid-spectrum autoimmune blistering disease but did not independently distinguish bullous pemphigoid (BP) from MMP. In accordance with consensus-based diagnostic principles, classification was therefore based primarily on the dominant clinical phenotype, the chronological sequence and extent of mucosal versus cutaneous disease, the number of involved mucosal sites, and the presence or risk of cicatricial damage (3, 4, 7–9). The long-standing oral disease, subsequent multisite mucosal involvement, nasal adhesions, visible symblepharon, and later generalized cutaneous eruption supported multisite MMP with extensive cutaneous involvement rather than BP with secondary mucosal lesions. Pemphigus was considered separately but was not supported because acantholysis and an intercellular direct immunofluorescence pattern were absent. The mildly increased desmoglein antibody levels were therefore interpreted as possible epitope spreading, chronic tissue injury, or nonspecific serological reactivity rather than concomitant pemphigus.

### Therapeutic intervention and follow-up

Given the patient’s advanced age, chronic obstructive pulmonary disease, recent weight loss with poor nutritional reserve, previous malignancy treatment, and persistent disease activity despite prolonged systemic glucocorticoid therapy, escalation to conventional broad immunosuppression was considered high risk. After detailed discussion and written informed consent, off-label dupilumab was initiated with a 600-mg subcutaneous loading dose followed by 300 mg every 2 weeks, together with prednisone. Prednisone was gradually tapered according to clinical response and discontinued at month 3, after which dupilumab was continued as maintenance monotherapy at the same dose and interval. Supportive management included wound care, topical anti-inflammatory therapy, screening for secondary infection, nutritional support, and multidisciplinary monitoring.

At the 1-month follow-up, pruritus and new blister formation had markedly decreased, cutaneous erosions had largely re-epithelialized, oral lesions had improved, and ocular dryness and tearing were reduced (Figures 1E–H). Anti-BP180, anti-BP230, anti-desmoglein 1, and anti-desmoglein 3 antibody levels were30.62, 42.17, 38.96, and 25.59 RU/mL, respectively, representing modest decreases from baseline. Total serum IgE declined from 125.62 to 87.53 IU/mL, whereas the absolute eosinophil count remained unchanged at 0.1 × 10^9/L at the 1-month follow-up. These biomarkers were not subsequently reassessed at regular intervals. The pre-existing conjunctival adhesion consistent with symblepharon remained unchanged, despite improvement in ocular symptoms. At the latest follow-up, 4 months after treatment initiation, the patient had received 8 dupilumab administrations, including the loading dose, and had remained off systemic glucocorticoids for 1 month while continuing dupilumab every 2 weeks. Body weight had recovered, the conjunctival adhesion had not progressed, and no serious infection, immediate hypersensitivity reaction, or other serious adverse event had occurred.

## Discussion

BP and MMP share key immunopathological features, including subepidermal blistering, linear IgG and/or C3 deposition along the basement membrane zone, and possible reactivity against BP180 and BP230. These findings establish a pemphigoid-spectrum disorder but do not independently distinguish BP from MMP. Classification therefore relies primarily on the dominant clinical phenotype, the sequence and extent of skin and mucosal involvement, the number of affected mucosal sites, and the presence or risk of scarring (3, 4, 7–9). BP is predominantly a pruritic cutaneous blistering disease, with mucosal involvement usually limited and non-scarring, whereas MMP is characterized by predominant or exclusive mucosal disease, often involving multiple sites and carrying a risk of irreversible ocular, airway, esophageal, or anogenital damage.

In this patient, painful oral erosions preceded generalized cutaneous blistering by approximately 2 years, followed by ocular symptoms with visible conjunctival adhesion consistent with symblepharon, nasopharyngeal disease with nasal adhesions, and hoarseness and breathing discomfort suggestive of upper-airway involvement. This chronology, multisite mucosal distribution, and objective cicatricial changes favored multisite MMP with extensive cutaneous involvement rather than BP with secondary mucosal lesions. Pemphigus was considered separately but was unlikely because acantholysis and intercellular immunoreactant deposition were absent. Extended antigen testing, particularly for anti-laminin 332 given the patient’s remote history of prostate cancer, would have refined antigen-specific classification (10), but was unavailable at our institution. Although this represents a limitation, it does not preclude the diagnosis of MMP, which is based primarily on clinicopathological correlation and direct immunofluorescence, while antigen-specific serology remains supportive and may be negative (3, 4, 8, 9).

The contribution of this report lies not in the use of dupilumab across the pemphigoid spectrum generally, because dupilumab is now approved in the United States for adult bullous pemphigoid and this therapeutic advance has been summarized in a recent update (6, 11, 20). Rather, its clinical relevance relates to its use in MMP, which remains an off-label indication and differs from classic bullous pemphigoid in its mucosal predominance, antigenic heterogeneity, scarring tendency, and risk of irreversible ocular or airway damage. Published experience with dupilumab in MMP remains limited. Isolated reports have described responses in Brunsting-Perry pemphigoid (12, 13), whereas Wang et al. reported an MMP patient whose cutaneous lesions improved but whose oral and eyelid involvement required additional methylprednisolone after 3 months (14). In the present patient, oral, ocular, suspected upper-airway, and extensive cutaneous disease improved during combined prednisone and dupilumab therapy, allowing prednisone withdrawal at month 3 and continuation of dupilumab monotherapy. This clinical course is informative but should not be interpreted as evidence of superiority or uniform efficacy across all mucosal sites.

The biological rationale for IL-4/IL-13 blockade in MMP includes potential effects on IgE-related autoimmunity, eosinophilic inflammation, pruritus, B-cell support, and tissue remodeling. Linear tissue-bound IgE has been detected in MMP and may correlate with disease activity (15, 16), while conjunctival IL-13 expression and its profibrotic effects on conjunctival fibroblasts support a potential role in ocular disease (17, 18). In this patient, early improvement occurred during concomitant prednisone and dupilumab treatment and therefore cannot be attributed to dupilumab alone. At the 1-month follow-up, pruritus and new blister formation had markedly decreased, cutaneous erosions had largely re-epithelialized, oral lesions had improved, and ocular dryness and tearing were reduced. Anti-BP180, anti-BP230, anti-desmoglein 1, and anti-desmoglein 3 antibody levels decreased from 35.83, 44.53, 47.77, and 26.64 RU/mL at baseline to 30.62, 42.17, 38.96, and 25.59 RU/mL, respectively. Total serum IgE declined from 125.62 to 87.53 IU/mL, whereas the absolute eosinophil count remained unchanged at 0.1 × 10^9/L. These biomarkers were not subsequently reassessed at regular intervals. The pre-existing conjunctival adhesion consistent with symblepharon did not reverse despite improvement in ocular symptoms, but no further progression was observed during the 4-month follow-up, suggesting control of active ocular surface inflammation without reversal of established fibrosis. At the latest follow-up, the patient had received 8 dupilumab administrations, including the loading dose, and had remained off systemic glucocorticoids for 1 month while continuing dupilumab every 2 weeks. His body weight had recovered, and no serious infection, immediate hypersensitivity reaction, or other serious adverse event had occurred. The main limitations were the short follow-up, the absence of standardized MMP disease activity scoring, and the lack of regular serial monitoring of BP180/BP230 antibodies, total IgE, and eosinophil counts beyond the 1-month reassessment. These limitations preclude robust evaluation of long-term treatment durability and biomarker–clinical correlations. Ocular monitoring remains essential because both ocular MMP and dupilumab-associated ocular surface disease may cause clinically significant morbidity (11, 19). Larger case series and prospective studies are needed to clarify patient selection, treatment sequencing, maintenance duration, relapse risk, and long-term safety.

## Data Availability

The original contributions presented in the study are included in the article/supplementary material, further inquiries can be directed to the corresponding authors.
